# *BRAF*, *KRAS *and *PIK3CA *mutations in colorectal serrated polyps and cancer: Primary or secondary genetic events in colorectal carcinogenesis?

**DOI:** 10.1186/1471-2407-8-255

**Published:** 2008-09-09

**Authors:** Sérgia Velho, Cátia Moutinho, Luís Cirnes, Cristina Albuquerque, Richard Hamelin, Fernando Schmitt, Fátima Carneiro, Carla Oliveira, Raquel Seruca

**Affiliations:** 1 Institute of Molecular Pathology and Immunology, University of Porto, Portugal; 2 Centro de Investigação de Patobiologia Molecular-CIPM, Instituto Português de Oncologia Francisco Gentil, Lisboa, Portugal; 3 Inserm, UMRS 762, 75010, Paris, France; 4 Faculty of Medicine, University of Porto, Portugal; 5 Hospital of S. João, Porto, Portugal

## Abstract

**Background:**

*BRAF*, *KRAS *and *PIK3CA *mutations are frequently found in sporadic colorectal cancer (CRC). In contrast to *KRAS *and *PIK3CA *mutations, *BRAF *mutations are associated with tumours harbouring CpG Island methylation phenotype (CIMP), *MLH1 *methylation and microsatellite instability (MSI). We aimed at determine the frequency of *KRAS*, *BRAF *and *PIK3CA *mutations in the process of colorectal tumourigenesis using a series of colorectal polyps and carcinomas. In the series of polyps CIMP, *MLH1 *methylation and MSI were also studied.

**Methods:**

Mutation analyses were performed by PCR/sequencing. Bisulfite treated DNA was used to study CIMP and *MLH1 *methylation. MSI was detected by pentaplex PCR and Genescan analysis of quasimonomorphic mononucleotide repeats. Chi Square test and Fisher's Exact test were used to perform association studies.

**Results:**

*KRAS*, *PIK3CA *or *BRAF *occur in 71% of polyps and were mutually exclusive. *KRAS *mutations occur in 35% of polyps. *PIK3CA *was found in one of the polyps. V600E *BRAF *mutations occur in 29% of cases, all of them classified as serrated adenoma. CIMP phenotype occurred in 25% of the polyps and all were mutated for *BRAF*. *MLH1 *methylation was not detected and all the polyps were microsatellite stable. The comparison between the frequency of oncogenic mutations in polyps and CRC (MSI and MSS) lead us to demonstrate that *KRAS *and *PIK3CA *are likely to precede both types of CRC. *BRAF *mutations are likely to precede MSI carcinomas since the frequency found in serrated polyps is similar to what is found in MSI CRC (*P *= 0.9112), but statistically different from what is found in microsatellite stable (MSS) tumours (*P *= 0.0191).

**Conclusion:**

Our results show that *BRAF*, *KRAS *and *PIK3CA *mutations occur prior to malignant transformation demonstrating that these oncogenic alterations are primary genetic events in colorectal carcinogenesis. Further, we show that *BRAF *mutations occur in association with CIMP phenotype in colorectal serrated polyps and verified that colorectal serrated polyps and MSI CRC show a similar frequency of *BRAF *mutations. These results support that *BRAF *mutations harbour a mild oncogenic effect in comparison to *KRAS *and suggest that *BRAF *mutant colorectal cells need to accumulate extra epigenetic alterations in order to acquire full transformation and evolve to MSI CRC.

## Background

In microsatellite unstable (MSI) colorectal tumours, both sporadic and inherited forms, hundreds of thousands of mutations accumulate within repetitive sequences throughout the genome [1]. Although genes with repetitive sequences are clear targets in tumours with a defective mismatch repair system, mutations in non-repetitive sequences are also found in MSI tumours. An example of this is the occurrence of activating mutations of *KRAS*, *PIK3CA *and *BRAF *genes.

Activating mutations of *BRAF *and *KRAS *are frequently found in sporadic colorectal (CRC) cancer. *BRAF *mutations occur in about 10 to 18% of CRC overall and in 30 to 45% of MSI CRC [2,3], more frequently in tumours harbouring *MLH1 *promoter hypermethylation [3] and with a CpG island methylation phenotype (CIMP) [4]. In microsatellite stable (MSS) CRC *BRAF *mutations are rare and whenever present are associated with advanced carcinomas [5]. *KRAS *mutations occur in both MSI (in about 20%) [3,6,7] and MSS (in about 35%) subsets of sporadic CRC [5,6]. Within the MSI subset of CRC *KRAS *mutations do not associate with the presence of *MLH1 *promoter hypermethylation [3] or with the presence of CIMP [8]. *PIK3CA *mutations were identified in various tumour models [9]. In colorectal tumours, *PIK3CA *mutations are present in 14% to 25% of the cases [9,10] and no differences in frequency and type of *PIK3CA *mutations were found between MSI and MSS subsets [10]. Further, whenever *PIK3CA *mutations occur in CRC they can occur in concomitance with activating *KRAS*-*BRAF *mutations, both in MSI and MSS tumours [10]. Nothing is known on the relationship between *PIK3CA *mutations and CIMP or *MLH1 *methylation in colorectal carcinomas.

The aim of the present study was to determine the timing of occurrence of *KRAS*, *BRAF *and *PIK3CA *mutations in the process of colorectal tumourigenesis and study the association of these mutational oncogenic events with CIMP, *MLH1 *methylation and MSI phenotype. In order to achieve our goal we evaluated the frequency and type of these epi/genetic events in a series of 17 colorectal polyps. Furthermore, we compared the frequency of *KRAS*, *BRAF *and *PIK3CA *mutations found in polyps with the frequency found in a series of 103 sporadic colorectal tumours, 50 MSI CRC and 53 MSS CRC, in order to understand the importance of these oncogenic events for the progression of the various types of colorectal cancer.

In conclusion, our results show that mutations of *BRAF*, *KRAS *and *PIK3CA *occur in non-malignant lesions of colorectum demonstrating that these oncogenic alterations are primary genetic events in colorectal carcinogenesis. Further, we show that *BRAF *mutations occur in association with CIMP phenotype in colorectal serrated polyps while *KRAS *mutations are found alone. These results support that *BRAF *mutations harbour a mild oncogenic effect in comparison to *KRAS *and suggest that *BRAF *mutant colorectal cells need to accumulate extra epigenetic alterations in order to acquire full transformation.

## Methods

Representative blocks of 17 colorectal polyps and 103 colorectal carcinomas formalin-fixed, paraffin embedded were retrieved from the Department of Pathology of the Hospital S. João. This study was a retrospective analysis and is in compliance with Helsinki declaration . The 103 sporadic CRC were previously classified for microsatellite status, 53 were MSS and 50 were MSI colorectal carcinomas [10]. The histological classification of the seventeen paraffin embedded colorectal polyps is listed in table 1. None of the patients included in this study had a positive family history. DNA from these 17 polyps was extracted using the Invisorb Spin Tissue Mini Kit (Invitek). *BRAF *(exon 15) and *KRAS *(exon 1) were amplified by PCR and the presence of mutations was detected by direct sequencing. To perform methylation studies, DNA extracted from paraffin embedded lesions was submitted to bisulfite treatment. CIMP was determined in 12 cases by methylation specific PCR (MSP) using the new panel of five markers described by Weisenberger [4]. The polyps were classified as CIMP (≥ 3 methylated markers), and CIMP-negative (≤ 2 methylated markers). We were able to analyze *MLH1 *methylation in 7 samples. For that the promoter region of the gene was amplified using a set of CpG island flanking primers (sense 5'ttttgtttttattggttggatattt, antisense 5'ccttcaaccaatcacctcaatacct) followed by direct sequencing. Microsatellite instability studies were performed by genescan analyses of a PCR amplified panel of five mononucleotide markers (NR27, NR24, NR21, Bat25 and Bat26) using the Multiplex PCR Kit (Qiagen). MSI was considered when two or more microsatellite markers were altered. Association studies between the frequency of *KRAS*, *PIK3CA *and *BRAF *in colorectal polyps and MSI and MSS CRC were performed using the Chi Square test and Fisher's Exact test. A *P*-value ≤ 0.05 was considered to be statistically significant.

**Table 1:** Summary of the molecular and histological features of the colorectal polyps analyzed

	**Molecular Classification**						**Histological classification**	
		
**Sample**	***KRAS***	***BRAF***	***PIK3CA***	**CIMP**	**MLH1 methylation**	**Microsatellite instability**	**Polyp type**	**Dysplasia**
1	G12D	-	-	Negative	UnMet	MSS	MHA	Mild
2	G12D	-	-	Negative	nd	MSS	MHA	Mild
3	G12V	-	-	nd	UnMet	MSS	MHA	Mild
4	G12V	-	-	Negative	nd	MSS	HP	-
5	G13D	-	-	Negative	nd	MSS	HP	-
6	G13D	-	-	Negative	nd	MSS	MHA	Mild
7	-	V600E	-	Positive	UnMet	MSS	SA	Mild
8	-	V600E	-	Positive	UnMet	MSS	SA	Mild
9	-	V600E	-	Positive	nd	MSS	SA	Mild
10	-	V600E	-	Negative	nd	MSS	SA	Mild
11	-	V600E	-	nd	nd	MSS	SA	Moderate
12	-	-	R1023Q	nd	nd	MSS	MHA	Mild
13	-	-	-	Negative	UnMet	MSS	MHA	Mild
14	-	-	-	Negative	UnMet	MSS	HP	-
15	-	-	-	Negative	UnMet	MSS	MHA	Mild
16	-	-	-	nd	nd	MSS	MHA	Mild
17	-	-	-	nd	nd	MSS	HP	-

UnMet – unmethylated; nd – not determined; MHA – Mixed hyperplastic and adenomatous; HP – Hyperplastic polyp; SA – Serrated adenoma

## Results

### *KRAS*, *PIK3CA*, and *BRAF *mutations occur prior to malignant transformation

In our series of colorectal polyps, we found that mutations in *KRAS*, *PIK3CA *or *BRAF *are mutually exclusive and occur in the majority of these pre-malignant colorectal lesions. Mutations in *KRAS*, *PIK3CA *or *BRAF *occur in 12 (70.6%) of the 17 colorectal polyps (Table 1).

*KRAS *mutations were found in 35.3% (6/17) of polyps. *KRAS *mutant polyps showed three different types of mutations with the same frequency within the two most common hotspot codons (12 and 13): G12D (33.3% – 2/6), G12V (33.3% – 2/6) and G13D (33.3% – 2/6). *KRAS *mutations were only found in polyps with some areas with dysplasia.

In contrast with the high frequency of *KRAS *mutations found in our series, *PIK3CA *mutations were observed in only one of the polyps analyzed (5.9%) (Table 1). The *PIK3CA *mutation found was localized to exon 20 and affects amino acid 1023 leading to an arginine-glutamine substitution (R1023Q). This *PIK3CA *mutation resides in a hotspot exon for mutations that has been previously reported in a MSI colorectal tumour [10].

*BRAF *mutations were found in 29.4% (5/17) of the cases (Table 1). All *BRAF *mutations found in our series of polyps corresponded to the hotspot *BRAF *V600E and all polyps with *BRAF *mutations exhibited the serrated architecture.

### *BRAF *mutations occur in association with CIMP phenotype in colorectal polyps

We studied the presence of CIMP phenotype, *MLH1 *promoter methylation and MSI phenotype and their association with the presence of *KRAS*, *BRAF *and *PIK3CA *mutations in the series of polyps.

Using the Weisenberger [4] panel of CIMP markers three of 12 polyps analyzed (25%) were classified as CIMP (≥ 3 methylated markers). All of the CIMP polyps were mutated for *BRAF *(Table 1). None of the CIMP polyps harbour *KRAS *mutations.

The methylation analysis of *MLH1 *was possible in 7 of the 17 polyps. Two of these 7 polyps were mutated for *KRAS*, 2 harboured *BRAF *mutations and 3 were wild-type for both genes. None of the 7 polyps studied harboured methylation in the promoter region of *MLH1 *(Table 1). Furthermore, we determined the MSI status of all polyps using the pentaplex set of microsatellite markers [11]. None of them were MSI.

### Colorectal serrated polyps and MSI CRC show a similar frequency of BRAF mutations

**Figure 1:**
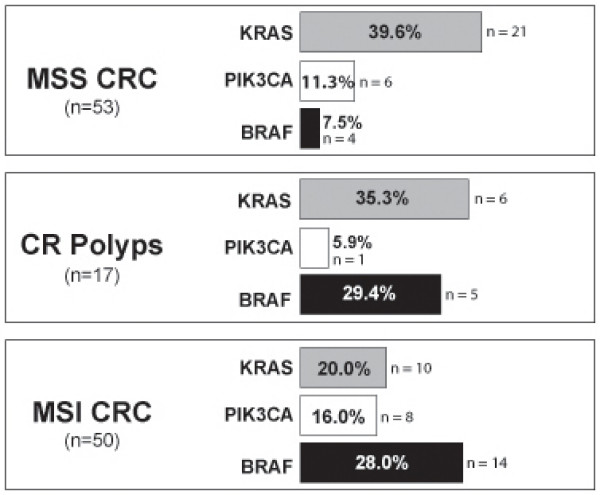
***KRAS*, *PIK3CA *and *BRAF *mutation frequencies between MSI/MSS CRC and CR polyps.** The frequency of *KRAS*, *PIK3CA *and *BRAF *mutations in MSI and MSS CRC was based on previous results of our group in Velho [10]. In brackets is represented the total of cases that were analyzed for each gene.

In order to understand the importance of *KRAS*, *PIK3CA *and *BRAF *mutations for the progression of the various types of colorectal cancer, we compared the frequency of these oncogenic events in the series of polyps and in 50 MSI and 53 MSS CRC (Figure 1).

Ten of the 50 (20%) MSI and 21 of the 53 (39.6%) MSS colorectal carcinomas exhibited *KRAS *mutations. The frequency of *KRAS *mutations found in the MSI and MSS CRC was not statistical different from the frequency found in colorectal polyps (*P *= 0.2014 and *P *= 0.7497, respectively).

The same was observed for *PIK3CA *mutations. MSI and MSS colorectal carcinomas harbour *PIK3CA *in 8 of the 50 (16%) and in 6 of the 53 (11.3%) cases, respectively. The frequency of mutations found in polyps, although lower in comparison to colorectal carcinomas, is not statistically different from MSI (*P *= 0.4296 - Fisher's Exact P-Value) and MSS (*P *> 0.9999 - Fisher's Exact P-Value) colorectal carcinomas.

Concerning the frequency of *BRAF *alterations in colorectal carcinomas, 14 of the 50 (28%) MSI and 4 of the 53 (7.5%) in MSS colorectal tumours show *BRAF *mutations. We found that MSI CRC and colorectal serrated polyps show a similar frequency of *BRAF *mutations (*P *= 0.9112). In contrast, the frequency of *BRAF *mutations in MSS carcinomas and colorectal polyps is significantly different (*P *= 0.0191).

## Discussion

Distinct types of colorectal polyps represent pre-malignant lesions of the intestinal tract. In our series of colorectal polyps we found the majority (71%) harbour mutations in *KRAS *(35.3%), *PIK3CA *(5.9%) or *BRAF *(29.4%) genes. Regarding *KRAS *mutations we show that these mutations occur in cases of hyperplasic polyps and in hyperplastic polyps with some areas of dysplasia in accordance to the recent reports of Wynter [12] and O'Brien [13] showing that *KRAS *mutations occur in pre-malignant lesions of the colorectum other than pure adenomas. When comparing the frequency of *KRAS *mutations found in our series of colorectal polyps with those observed within the MSS and MSI subsets of CRC, the frequency of *KRAS *mutations found in polyps is not statistically different from the frequency observed in MSI and MSS colorectal tumours (Figure 1). Both observations highlight the role of *KRAS *activation in the initiation of the different types of sporadic CRC [12-14] besides its role in the adenoma-carcinoma multistep pathway of colorectal carcinogenesis [15].

*PIK3CA *mutations were found in only one of the polyps (6%) but this frequency is not significantly different from what is observed in CRC independently of MSI status. The mutation found in our series of colorectal polyps was previously described in a colorectal tumour [10] suggesting that it harbours a pathogenic effect. Further, it is interesting to note that the *PIK3CA *alteration found in the single affected polyp is wild-type *KRAS *and *BRAF *case. This result suggests that mutant *PIK3CA *is likely to be an alternative rare genetic event to *KRAS *and *BRAF *in pre-malignant lesions of the colon.

The frequency of *BRAF *mutations in polyps (30%) is higher than the frequency observed in overall CRC (17%). This observation lead us to speculate that a fraction of *BRAF *mutant polyps do not progress to carcinoma and remain as non-malignant colorectal lesions as an endpoint stage. In accordance with this, it was demonstrated in mice that expression of active *BRAF *in the lungs lead to an initial burst of cell proliferation followed by growth arrest rarely inducing spontaneous progression to adenocarcinoma unless mice were deliberately engineered to lack the *TP53 *or *Ink4a/Arf *tumour suppressor genes [16]. This study and our data suggest that *BRAF *may need to cooperate with other factors (genetic or epigenetic) to progress to colorectal carcinoma, as it was previously proposed for human naevi [17,18].

Noteworthy, the frequency of *BRAF *mutations in colorectal polyps is similar to the frequency observed in MSI CRC (28%) (*P *= 0.9112). These data raises the hypothesis that mutant *BRAF *triggers a distinct pathway of colorectal carcinogenesis leading to the formation of MSI colorectal tumours different from the adenoma-carcinoma sequence. Interestingly *BRAF *mutations occurred only in polyps with the serrated architecture supporting that *BRAF *activation is pivotal in the serrated pathway of CRC as advanced previously by Jass [19,20] and other authors [13,14,21,22].

Analyzing the frequency of CIMP in colorectal polyps we have demonstrated that the results obtained are similar to what was described in CRC [13,14,20,22] showing that *BRAF *mutations are associated with CIMP phenotype in the initial steps of carcinogenesis before malignant transformation. In contrast, *KRAS *mutations were only associated to with low levels of methylation as it was previously observed in primary colorectal tumours [19,23], demonstrating that *KRAS *mutations are associated to mild effects in the methylation profile during colorectal carcinogenesis. These results support that *BRAF *mutations harbour a mild oncogenic effect in comparison to *KRAS *and suggest *BRAF *mutant colorectal cells need to accumulate extra epigenetic alterations in order to acquire full transformation.

MSI and loss of expression of MLH1 due to promoter hypermethylation are frequently associated with *BRAF *mutation and CIMP in MSI sporadic colorectal tumours [2-4]. In our series of colorectal polyps we did not detected *MLH1 *promoter hypermethylation which is in agreement with the fact that none of the analyzed polyps harboured MSI. Our data is in agreement with the results previously described in serrated adenomas described by Sawyer [24] demonstrating that *BRAF *and CIMP precedes MSI as proposed by the model advanced by O'Brien [14]. The late acquisition of *MLH1 *methylation leading to the accumulation of mutations throughout the genome targeting important genes (tumour suppressor genes or proto-oncogenes) involved in the control of cellular homeostasis may bridge the transition from a pre-malignant to a malignant lesion.

## Conclusion

In conclusion, our results show that mutations of *KRAS *and *PIK3CA *occur in non-malignant lesions of colorectum and the frequency of these mutations is not distinct in polyps and MSS and MSI CRC demonstrating that these oncogenic alterations are primary genetic events in the two subsets of colorectal carcinogenesis. Further, we show that *BRAF *mutations occur in association with CIMP phenotype in colorectal serrated polyps while *KRAS *mutations are found alone. Further we show that only MSI CRC and colorectal serrated polyps harbour similar frequency of *BRAF *mutations. These results support that *BRAF *mutations harbour a mild oncogenic effect in comparison to *KRAS *and suggest that *BRAF *mutant colorectal cells need to accumulate extra epigenetic alterations in order to acquire full transformation and evolve to MSI CRC.

## Competing interests

The authors declare that they have no competing interests.

## Authors' contributions

SV did *BRAF *and *PIK3CA *mutation screening, performed methylation studies (CIMP and MLH1 methylation) and drafted the manuscript. CM did *KRAS *mutation screening. LC performed MSI analyses. CA collected patient material with distinct subtypes of colorectal polyps. RH analyzed the MSI status of CRC. FS and FC were responsible for the histological classification of CRC. CO did BRAF and *KRAS *screening in CRC and helped to draft the manuscript. RS conceived and designed the study, analyzed the results and helped to draft the manuscript. All authors read and approved the final manuscript.

## Pre-publication history

The pre-publication history for this paper can be accessed here:


